# Allele-biased expression of the bovine *APOB* gene associated with the cholesterol deficiency defect suggests cis-regulatory enhancer effects of the LTR retrotransposon insertion

**DOI:** 10.1038/s41598-022-17798-5

**Published:** 2022-08-05

**Authors:** Doreen Becker, Rosemarie Weikard, Annika Heimes, Frieder Hadlich, Harald M. Hammon, Marie M. Meyerholz, Wolfram Petzl, Holm Zerbe, Hans-Joachim Schuberth, Martina Hoedemaker, Marion Schmicke, Susanne Engelmann, Christa Kühn

**Affiliations:** 1 Institute of Genome Biology, Research Institute for Farm Animal Biology (FBN), Dummerstorf, Germany; 2 Institute of Nutritional Physiology, Research Institute for Farm Animal Biology (FBN), Dummerstorf, Germany; 3 Clinic for Ruminants with Ambulatory and Herd Health Services, Centre for Clinical Veterinary Medicine, Ludwig-Maximilians-University Munich, Oberschleißheim, Germany; 4 Institute for Immunology, University of Veterinary Medicine Hannover, Hannover, Germany; 5 Clinic for Cattle, University of Veterinary Medicine Hannover, Hannover, Germany; 6 Faculty of Natural Sciences III, Martin-Luther University Halle-Wittenberg, Halle, Germany; 7 Institute for Microbiology, Technical University Braunschweig, Brunswick, Germany; 8 Microbial Proteomics, Helmholtz Centre for Infection Research, Brunswick, Germany; 9 Agricultural and Environmental Faculty, University of Rostock, Rostock, Germany

**Keywords:** Genetics, Molecular biology, Physiology, Pathogenesis

## Abstract

The insertion of an endogenous retroviral long terminal repeat (LTR) sequence into the bovine apolipoprotein B (*APOB*) gene is causal to the inherited genetic defect cholesterol deficiency (CD) observed in neonatal and young calves. Affected calves suffer from developmental abnormalities, symptoms of incurable diarrhoea and often die within weeks to a few months after birth. Neither the detailed effects of the LTR insertion on *APOB* expression profile nor the specific mode of inheritance nor detailed phenotypic consequences of the mutation are undisputed. In our study, we analysed German Holstein dairy heifers at the peak of hepatic metabolic load and exposed to an additional pathogen challenge for clinical, metabolic and hepatic transcriptome differences between wild type (CDF) and heterozygote carriers of the mutation (CDC). Our data revealed that a divergent allele-biased expression pattern of the *APOB* gene in heterozygous CDC animals leads to a tenfold higher expression of exons upstream and a decreased expression of exons downstream of the LTR insertion compared to expression levels of CDF animals. This expression pattern could be a result of enhancer activity induced by the LTR insertion, in addition to a previously reported artificial polyadenylation signal. Thus, our data support a regulatory potential of mobile element insertions. With regard to the phenotype generated by the LTR insertion, heterozygote CDC carriers display significantly differential hepatic expression of genes involved in cholesterol biosynthesis and lipid metabolism. Phenotypically, CDC carriers show a significantly affected lipomobilization compared to wild type animals. These results reject a completely recessive mode of inheritance for the CD defect, which should be considered for selection decisions in the affected population. Exemplarily, our results illustrate the regulatory impact of mobile element insertions not only on specific host target gene expression but also on global transcriptome profiles with subsequent biological, functional and phenotypic consequences in a natural in-vivo model of a non-model mammalian organism.

## Introduction

In 2015, a genetic defect termed Cholesterol Deficiency (CD, https://omia.org/OMIA001965/9913/) was described in the worldwide Holstein Friesian cattle population^1^. CD-affected neonatal calves suffered from insufficient development in combination with symptoms of incurable diarrhoea and generally died within the first 6 months after birth^1^. Concentrations of cholesterol and triglycerides in the blood were markedly reduced in affected individuals, indicating a disorder of lipid metabolism^2–5^. Pedigree analysis of affected calves initially suggested an autosomal recessive mode of inheritance^1, 5^, and the insertion of a transposable long terminal repeat (LTR) element in exon 5 of the *apolipoprotein B* (*APOB*) gene was found as causal mutation^5–7^. The encoded APOB protein is a crucial key component for the transport of lipid molecules, including cholesterol, through the body and into cells. Initial results indicated that the insertion comprised a 1299 bp solo-long terminal repeat (LTR) insertion, which was predicted to result in a premature stop codon causing a protein that is 97% truncated compared to its wild-type sequence. Causal mutation data were later updated by a report describing that indeed a full-length bovine endogenous retroviral K element (ERV-K) had integrated into the coding part of the gene at position 11:77,891,739^8^. The ERV-K was flanked by two identical full-length LTRs, and the open reading frames within the retroposon genes were altered (loss-of-function)^8^. Menzi et al.^7^ provided data that indicates a complete lack of transcribed sequence for the *APOB* gene downstream of the LTR insertion, and Harland^8^ reported a polyadenylation signal in the 5′ERV-LTR, which presumably generates a premature poly(A) tail and subsequent termination of transcription. ERV-LTRs are known to exert regulatory activities as reviewed by Chuong et al.^9^, and due to their autonomous promoters, they can generate chimeric transcripts and readthrough transcription^10^. However, many studies on the regulatory effects of LTRs are based on bioinformatic prediction from functional genomic studies or on CRISPR-mediated modification of (cancer) cell lines. There are few studies on the effects of LTRs in their natural in-vivo context^9^.

In addition to its molecular genetic background, also pathophysiological consequences of the CD mutation are controversially discussed^11–14^. Recent data challenge the recessive inheritance mode of CD. Häfliger et al.^13^ suggested that the disorder is most likely dominantly inherited with incomplete penetrance in heterozygous CDC carriers (nomenclature defect according to the World Holstein Friesian Federation, http://www.whff.info/documentation/documents/GeneticTraitsandCarrierCodes.pdf). But although Gross et al.^12^ found differences in cholesterol and lipoprotein concentrations between CDC and homozygous wild type (CDF) animals, CDC and CDF cows did not differ in other metabolic parameters, milk yield or fertility. Furthermore, the question arose, whether cholesterol synthesis and turnover are indeed impaired as a consequence of the CD mutation or whether rather the pathophysiological effect of the mutation is only induced via malabsorption and impaired transportation of lipid components due to a lack of APOB to form lipoprotein complexes^12^.

Thus, to explore the molecular consequences of the LTR insertion on *APOB* expression and subsequent effects on lipid metabolism, we selected an experimental design, which presented a substantial challenge to APOB regulation and lipid metabolism. The lipid metabolism of cows is particularly critical in the first weeks after parturition due to a severe negative energy balance associated with a substantial lipomobilization^15^, and even more so in situations of pathogen-challenges^16^. Thus, to explore the molecular regulatory consequences of the ERV-LTR insertion on *APOB* expression and subsequent effects on lipid metabolism, our study monitored hepatic expression pattern and biochemical phenotypes of CDC and CDF early lactation cows after an intramammary challenge with relevant mastitis pathogens (*Staphylococcus (S.) aureus* or *Escherichia (E.)* *coli*).

## Material and methods

### Selection of animals

The cows investigated in this study were part of a large network project to evaluate genetic predisposition for disease susceptibility in Holstein Friesian cows. Details on the selection and management of the animals have been previously described^17–20^. The final cohort comprised 35 animals, seven heterozygous CDC carriers and 28 homozygous wild type CDF animals. Homozygous mutant CDS cows were not available, presumably due to the well-described fatal effects of the mutation in early life. The selected heifers were purchased from dairy cow farms across Germany prior to first calving and were brought to the Clinic for Cattle at the University of Veterinary Medicine Hannover (TiHo) for a highly standardized challenge experiment. Only CDC cows were recruited that did not show manifest clinical symptoms of the CD defect before first calving (e.g., poor development, diarrhoea unresponsive to treatment^4^).

For the cow cohort, the experiment was performed under the reference number 33.12-42502-04-15/2024 approved by the Lower Saxony Federal State Office for Consumer Protection and Food Safety. This study was also submitted to and approved by the ethics committees of the Leibniz Institute for Farm Animal Biology and the University of Veterinary Medicine Hanover, respectively. An independent previous calf experiment had been conducted at the Educational and Research Centre for Animal Husbandry, Hofgut Neumuehle, Germany. It had been permitted by the local department for animal welfare affairs (Landesuntersuchungsamt, Koblenz, Germany) (23 177-07/G 13-20-069). All ethical evaluations were performed as required by the German Animal Care law and associated legislative regulations^21^. The reporting in the manuscript follows the recommendations in the ARRIVE guidelines. This includes a comprehensive description of experimental animals and experimental procedures as provided in^18, 20^.

### Challenge experiment

The 35 animals were challenged in an intramammary infection model essentially as described in detail by Rohmeier et al.^20^. At 36 ± 3 days after parturition, 24 healthy cows (four CDC, 20 CDF) were challenged with 10,000 CFU (colony forming units) *S. aureus*_1027_ each in both hindquarters of the mammary gland. Eleven healthy cows (four CDC, eight CDF) were challenged with 500 CFU *E. coli*_1303_ in one hindquarter of the mammary gland^22^. A control udder quarter of each cow was infused with sterile sodium chloride solution.

### Clinical examination

Before and after the experimental challenge, cows were comprehensively monitored for clinical and subclinical diseases as described previously^18–20^. Particular attention was paid to signs of inflammation as well as to biochemical indicators of impaired energy and fat metabolism. To this end, blood serum concentration of beta-hydroxybutyrate (BHB) (mmol/L) and nonesterified fatty acids (NEFA; μmol/L) and blood plasma concentrations of insulin and IGF-1 were determined in weekly intervals starting 3 weeks before parturition until the end of the experiment with commercial kits as described by Meyerholz et al. ^19^ in 3 days intervals starting 3 weeks before parturition until the end of the experiment. Measuring plasma GH concentration was performed with an enzyme-linked immunosorbent assay^23^ with modifications and adaptations according to Kawashima et al.^24^ and Meyerholz et al.^25^.

For statistical data analyses, the GLIMMIX procedure of SAS 9.4.1 (SAS Institute Inc., Cary, NC) was applied. Prior to evaluation, we log10-transformed NEFA, BHB, insulin, GH and IGF-1 data. For milk yield, we considered energy-corrected milk as described by Meyerholz et al.^19^. All statistical analyses were performed using SAS 9.4.1. The general linear mixed model implemented in the GLIMMIX procedure included fixed effects for day and group, the interaction of day and group and a random sire effect G: y = β0 + β1∙day + β2∙group + β3∙day x group + G. For the effect of day, we had eight categories for days relative to parturition: − 18 ± 3; − 11 ± 3; − 4 ± 3; 1; 4 ± 3; 11 ± 3; 18 ± 3; 25 ± 3, 32 ± 3, the group comprised either CDC or CDF. Furthermore, covariance structure comprised the repeated subject cow in compound symmetry. For those data measured before and after calving, separate models were calculated.

At the end of the experiment (*S. aureus*: 96 h after infection; *E. coli*: 24 h after infection), the animals were stunned with a penetrating captive bolt pistol, immediately followed by exsanguination via longitudinal section of the jugular veins and carotid arteries. Liver tissue from the *Lobus caudatus* and mammary gland tissue were immediately shock frozen in liquid nitrogen and subsequently stored at − 80 °C.

### Transcriptome analysis by RNA sequencing

RNA isolation from liver was conducted as described by Heimes et al.^26^. A second DNase digestion step was added if contamination with genomic DNA was detected by PCR^27^. Repeatedly, RNA concentration and purity were measured on a NanoDrop 2000 spectrophotometer (Thermo Fisher Scientific, Waltham, MA) and a Qubit 2.0 fluorometer (Thermo Fisher Scientific, Waltham, MA). RNA integrity was quantified on the Bioanalyzer 2100 (Agilent Technologies, Böblingen, Germany). Finally, a strand-specific RNAseq library preparation protocol with poly(A) selection (TruSeq Stranded mRNA LP, Illumina, San Diego, CA) was applied to the samples. The RNAseq libraries were again monitored for quality on the Bioanalyzer 2100 and were paired-end sequenced on the Illumina HiSeq 2500 system (Illumina, San Diego, CA) for two times 90 base pairs.

For data management, Linux, and R scripts^28^ and SAMtools^29^ were used. Quality of the raw reads was checked with FastQC version 0.11.5^30^ and MultiQC version 1.4^31^. Subsequently, adapters were clipped with Cutadapt version 1.12^32^, and low qualities bases were eliminated with QualityTrim^33^. The reads were aligned to the bovine reference genome ARS1.2 with Ensembl 95 reference annotation with Hisat2 version 2.1.0^34^. The featureCounts option of the subread package version 1.6.2^35^ was used for strand-aware read counting. For differential expression analysis in liver samples, we applied DESeq2 version 1.26.0^36^ in a model fitting the pathogen used for the challenge and the carrier status at the CD locus as fixed effects. Finally, lists of differentially expressed genes (DEGs) were used for enrichment analyses in DAVID^37^ and Ingenuity Pathway Analysis (IPA, Qiagen, Hilden, Germany) for the identification of significantly enriched GO terms, KEGG and IPA pathways and predicted IPA upstream regulators with a threshold for significance of p_adj_ < 0.05.

### APOB expression analysis

For all individuals, read counts were determined for all *APOB* gene exons separately as well as combined for exons 1–4 (exons upstream of the LTR insertion site) and exons 5–29 (within and downstream of the LTR insertion site) using the featureCounts option of the subread package (version 1.6.2)^35^. The resulting counts were normalized for transcriptome-wide total read counts for each animal and the length of the respective exons. The resulting normalized read counts were screened for differences between CDC and CDF animals in a linear model using the lm function of the R packages lme4 (v. 1.1-25) and lsmeans (v.2.30-0). The fixed effect of pathogen type used for the challenge (*S. aureus, E. coli*) as well as the fixed effect of the *APOB* genotype (CDC, CDF) were fitted into the model.

To evaluate, whether both *APOB* haplotypes contributed equally to the increased *APOB* exon 1–4 expression in CDC animals, or whether there was an allelic imbalance, we took advantage of SNPs in exon 3 at position 77,887,611 and exon 13 at position 77,899,208 on BTA11 (NC_037338.1) identified after visual inspection of aligned reads via the Integrative Genomics Viewer (IGV, v2.7.2). To enable unbiased genotyping at the variant positions, genomic DNA was isolated from liver and mammary gland samples. From those samples, a genomic fragment including the entire exon 3 as well as a genomic fragment spanning exon 13 were amplified by PCR for genotyping the SNPs at positions 77,887,611 and 77,899,208, respectively. PCR amplification from genomic DNA was conducted with primers listed in Supplementary Table 1. The amplified PCR fragments were sequenced with the primers used for PCR amplification in forward and reverse directions. The obtained sequence profiles were aligned to the *APOB* mRNA (XM_024999521.1) and genomic (NC_037338.1) reference sequences and were visually inspected with Bioedit v7.2.5.0 to obtain the animals’ genotypes for the respective positions.

**Figure 1:**
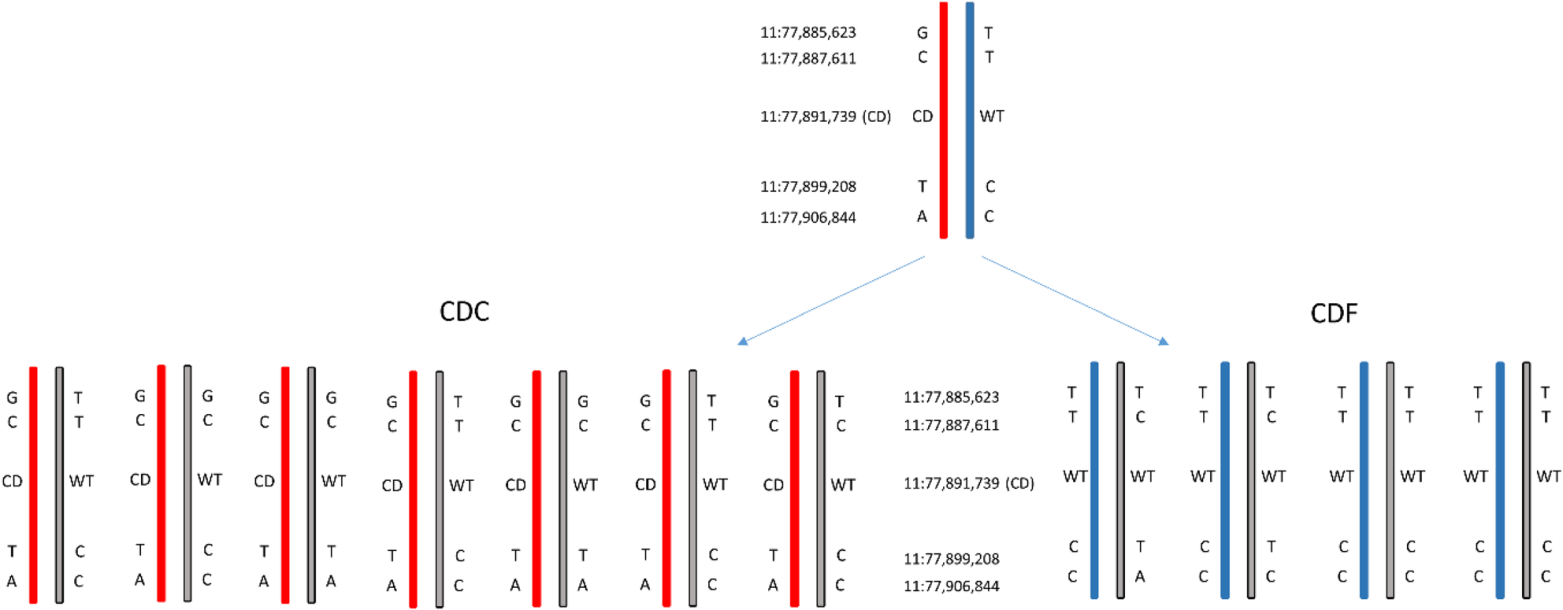
Identification of a haplotype carrying the CD mutation. Haplotype distribution of seven CDC and four CDF half-sib offspring of a CDC carrier sire for the chromosomal region on BTA11: 77,885,623–77,906,844 comprising the site of LTR retrotransposon insertion in the *APOB* gene causal for the CD defect. CD denotes the mutated allele of the *APOB* gene at position 11:77,891,739, WT denotes the wild type allele.

Furthermore, all 35 animals were genotyped with the Illumina Bovine HD chip (Illumina, San Diego/USA) comprising 777,692 SNPs to establish genotypes for the genomic target region on BTA11 upstream, downstream and within the *APOB* locus. SNPs in the target region were filtered for minor allele frequency (MAF > 0.05), deviation from Hardy–Weinberg equilibrium (*p* > 0.05) and GenTrain Score (GT > 0.7). Due to the fact, that some CDF and CDC heifers had a common sire, which had a confirmed CDC carrier status, we were able to conclude on the haplotype phases of the sire and the inherited haplotypes of the respective offspring within the *APOB* gene (see Fig. 1). Due to the very small genomic distance between exon 3 and exon 13, we assumed that double recombination events would be extremely unlikely. The haplotype analysis showed that the mutated CD allele was on the same haplotype as the “C” allele at position 77,887,611 in exon 3 and the “T” allele at position 77,899,208 in exon 13.

This information was used for an allele biased differential expression analysis for the positions 11:77,887,611 and 11:77,899,208. To this end, we established the total number of reads spanning position 11:77,887,611, which carried either the non-reference allele “C” or the reference allele “T” by extracting read coverage information from samtools mpileup^38^ (version 1.8) with subsequent Linux scripts. An analogous analysis was done for position 11:77,899,208 by calculating the reads covering the non-reference allele “T” or the reference allele “C”. We calculated the ratio of reads with the two alternative alleles (C to T for 77,887,611, T to C for 77,899,208) for both positions. Across all animals with a heterozygous genotype C/T at 77,887,611 or 77,899,208, we tested, if this ratio differed between CDC and CDF animals with a simple linear model fitting the CD genotype as a fixed effect. Furthermore, a t-test conducted in R (version 3.6.2) was applied to test whether the ratio differed significantly from the expected value of 1.

### Whole genome resequencing

To obtain further sequence information for the *APOB* region, genomic DNA from two CDC and CDF animals from the pathogen challenge experiment as well as two CDC calves from a previous independent experiment unrelated for at least three generations^39^ were used for whole genome resequencing (Novogene, Bejing, China) after PCR free library preparation on a NovaSeq flowcell with 2 × 150 bp sequencing. The reads obtained were processed according to the protocol for the 1000 bulls genome project^40^. This comprised quality trimming with Trimmomatic (v. Trimmomatic-0.38) and alignment to the bovine genome assembly ARS1.2^41^ with bwa^42^ and read sorting via samtools (version 1.8). PCR duplicates were marked and removed via Picard tools (Picard Toolkit. 2018. Broad Institute, GitHub Repository, http://broadinstitute.github.io/picard/; Broad Institute, version). GATK^43^ (GenomeAnalysisTK-3.8-1-0-gf15c1c3ef) was used for base recalibration (option BaseRecalibrator) and variant calling (option HaplotypeCaller). The respective pipeline code is provided as Supplementary File Text 1. The resulting genotype files were further processed with PLINK (version 1.9,^44^), bcftools (^29^ version 1.14) and in-house Linux scripts. In addition, we had available a further Holstein x Charolais F_2_ individual with hepatic transcriptome data^45^ (SAMEA6031983, PRJEB34570) and whole genome sequence data (1000 bulls genome project,^46^, Run 7), which also had a CDF wild type status.

To exclude other variants on the affected haplotype that could be causative for the allele-biased expression of the *APOB* gene alternative to the LTR retrotransposon insertion, we screened the region 500 kb upstream of the *APOB* gene LTR insertion. To this end, we filtered SNPs and small indels on BTA11 from position 11:77,385,988 to 77,891,739 bp (NC_037338.1) in the six resequenced animals using bcftools version 1.9^29^. We postulated that all CDC animals should be heterozygous for the variant causal for generating the haplotype-biased gene expression, whereas the variant should be homozygous for the CDF animals of the dataset because there was no indication of an allele-biased *APOB* expression in CDF animals.

## Results

### Differential hepatic transcriptome expression in CDC carrier cows

The hepatic transcriptome analysis by RNAseq generated 3.8 billion reads (Ø 109 million reads per sample). RNAseq datasets are submitted to the ENA repository (https://www.ebi.ac.uk/ena, Project number PRJEB33849, accession numbers ERR3466640-ERR3466680) at EMBL-EBI. Across all samples, on average 92.1% of reads mapped at least once to the reference genome ARS-UCD1.2 with Ensembl genome annotation release 95^47^. The data analysis revealed a total of 13,213 annotated genes expressed with an FPKM > 1 in at least four samples, which were subjected to subsequent differential expression analysis.

#### Differentially expressed genes

The whole transcriptome analysis revealed substantial differences in the hepatic transcriptome between the CDC and CDF groups and confirmed the observed phenotypic differences, particularly in lipid metabolism (see below). The expression analysis revealed 146 significantly (p_adj_ < 0.05) differentially expressed genes (DEGs) between CDC cows and CDF cows (Table 1 and Supplementary Table 2). 118 genes were downregulated and 28 upregulated in CDC cows compared to CDF cows.

**Table 1:** Top 10 significantly DEGs (sorted by p_adj_, ascending) in the differential expression analysis CDC cows versus CDF cows.

Gene symbol	Gene name	log_2_FoldChange	p_adj_
*APOB*	Apolipoprotein	− 0.95	5.29E−09
*GSTM1, ENSBTAG00000037673*	Glutathione S-Transferase Mu 1	− 1.83	5.16E−05
*CYP3A5*	Cytochrome P450 Family 3 Subfamily A Member 5	− 1.38	8.18E−05
*GSTM1, ENSBTAG00000017765*	Glutathione S-Transferase Mu 1	− 2.29	8.18E−05
*GSTM3*	Glutathione S-Transferase Mu 3	− 1.37	0.0015
*SULT2A1*	Sulfotransferase Family 2A Member 1	− 0.61	0.0024
*EBPL*	Emopamil Binding Protein Like	− 1.00	0.0027
*ELOVL6*	ELOVL fatty acid Elongase 6	− 1.29	0.0041
*CYP7A1*	Cytochrome P450 Family 7 subfamily A member 1	− 3.08	0.0047
*DBI*	Acyl-CoA-binding protein	− 0.90	0.0053

**Figure 2:**
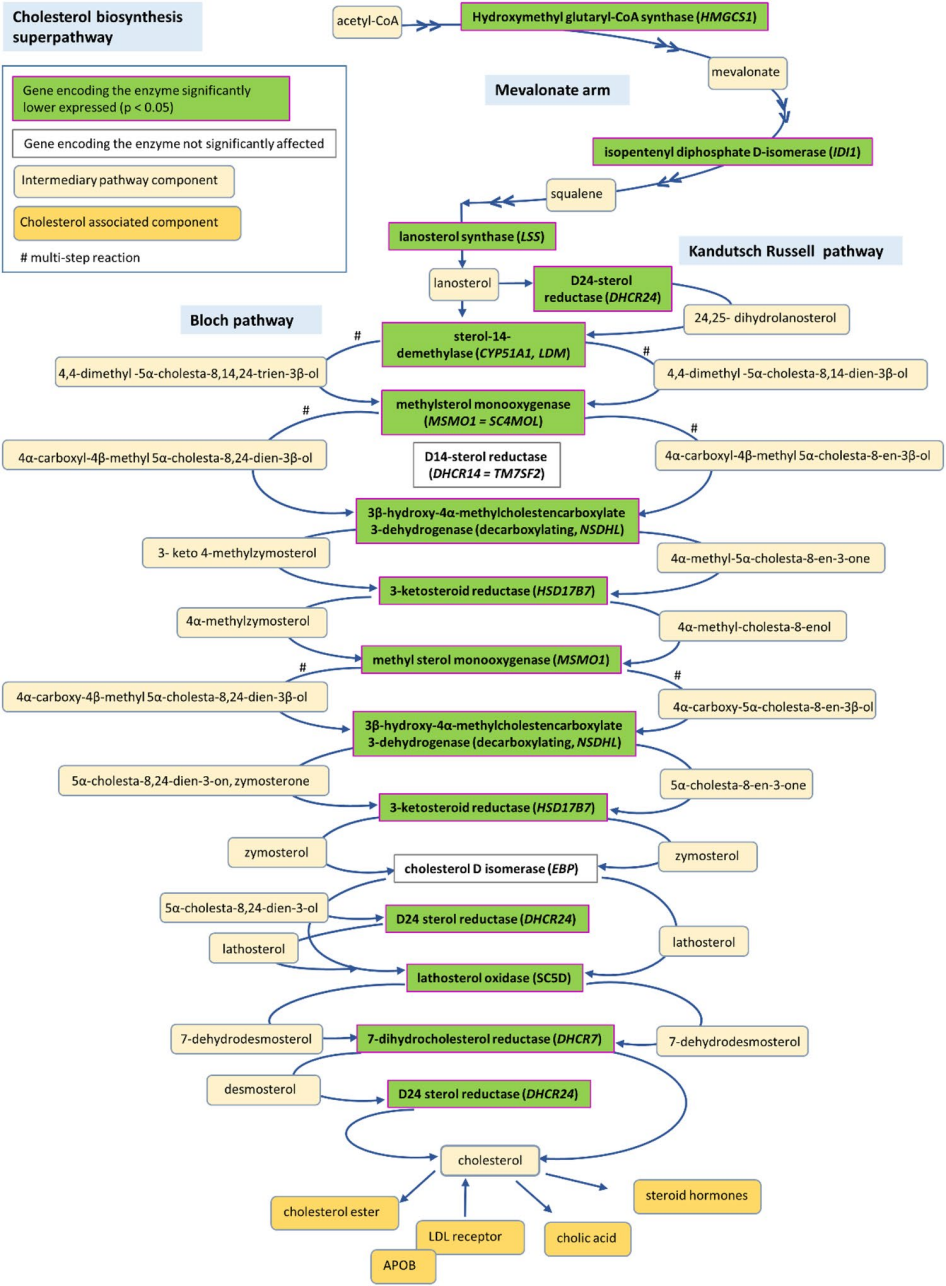
Ingenuity pathway *Cholesterol Biosynthesis Superpathway* significantly enriched of genes differentially expressed in the hepatic transcriptome between CDC and CDF cows.

On top of the DEGs was the *APOB* gene, which was found to be significantly lower expressed in CDC cows compared to CDF cows in the global transcriptome analysis (Table 1). As expected under the hypothesis of one gene dosage missing, the *APOB* expression in the CDC heterozygous carriers was about half (log_2_ fold change − 0.95, p_ajd_ = 5.3 × 10^–09^) of the expression in the CDF cows. Other gene members of the APO lipoprotein family, namely *APOA2* and *APOA4*, were also statistically significantly lower expressed in CDC heterozygous carriers than in wild-type animals (Supplementary Table 2). In addition to the APO lipoprotein family genes, further genes related to lipid metabolism were differentially expressed between CDC and CDF cows (particularly *ELOVL6, DBI* in Table 1 and Supplementary Table 2), but also e.g. *BDH2* (Hydroxybutyrate Dehydrogenase 2), *FADS1, FADS2* (Fatty Acid Desaturase 1 and 2), and *SCD (*Stearyl CoA desaturase) (Supplementary Table 2). Not only genes directly involved in lipid metabolism, but also genes involved in cholesterol biosynthesis displayed a significantly differential expression in CDC compared to CDF cows (Fig. 2). Examples in this context are *DHCR24* encoding 24-Dehydrocholesterol Reductase and *HSD17B7* encoding Hydroxysteroid 17-Beta Dehydrogenase 7, which are responsible for the last steps of the cholesterol synthesis (Supplementary Table 2). Also noteworthy is *CYP7A1*, because it encodes the enzyme for a rate-limiting step in cholesterol catabolism and bile acid biosynthesis. This gene displayed the highest fold change in the expression level of all genes between both haplotype groups (Table 1). All respective genes consistently displayed a lower expression in CDC heterozygote carriers compared to CDF homozygous wild type animals (Supplementary Table 2) showing that the LTR insertion is associated with significantly altered hepatic expression.

#### Pathways and GO terms enriched for differentially expressed genes

Enrichment analysis with the Database for Annotation, Visualization and Integrated Discovery (DAVID) investigating the collection of genes differentially expressed between CDC and CDF animals indicated seven enriched GO terms, 16 enriched KEGG pathways and seven REACTOME pathways (Supplementary Table 3).

The KEGG pathway Steroid hormone biosynthesis (Supplementary Figure 1) highlighted a number of DEGs, which encode key enzymes for the synthesis of key steroid hormones, e.g. cortisone, androstenone or estradiol 17β. The relevance of the CD defect allele for cholesterol biosynthesis is also confirmed by the top enriched Biological Process GO term cholesterol biosynthetic process. Furthermore, DEGs contained in the KEGG PPAR signaling pathway underlined the phenotypic observations that indeed the lipid metabolism is affected by the CD defect allele in CDC animals.

The Ingenuity pathway analysis identified 80 pathways significantly enriched with genes differentially expressed between CDC and CDF cows (Supplementary Table 4). Many of the highly enriched pathways are part of the lipid metabolism, and are specifically involved in cholesterol (Fig. 2) and steroid biosynthesis, which is in line with the data obtained from KEGG pathway analysis. The LPS/IL-1 Mediated Inhibition of RXR Function pathway involving the decoy IL1 receptor-encoding gene *IL1R2* that showed increased expression in CDC animals, also supported evidence for impaired cholesterol synthesis, because the DEGs in this pathway are predominantly linked to cholesterol, steroid and lipid synthesis (e.g. *CYP2C19*).

The analysis of potential upstream regulators predicted a list of more than 400 potentially regulating molecules (Supplementary Table 5). However, analogously to the pathway enrichment analysis, the most significant potential regulators are metabolites, receptors or transcription regulators known for their role in lipid metabolism.

### APOB differential expression analyses

**Figure 3:**
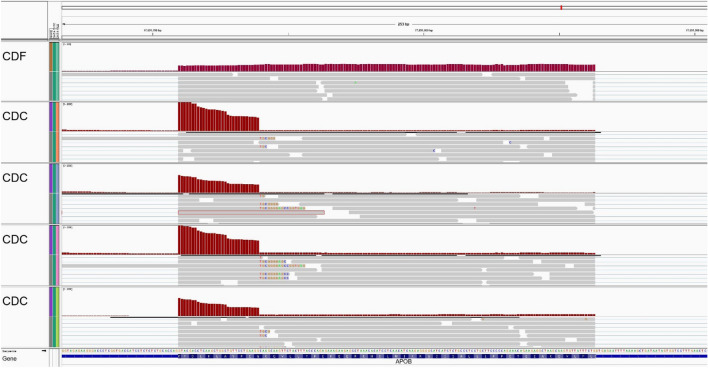
Integrative Genomics Viewer screenshot of *APOB* exon 5 liver expression profiles. At the top an unaffected CDF animal, below four CDC cows (note the different y-axis scales).

To gain further insight into the consequences of structural alterations of the *APOB* gene in CDC carrier animals, we took a closer look at the respective chromosomal region (BTA11: 77,885,935–77,927,967 bp ^7^) with the Integrative Genomics Viewer version 2.7.2^48^. As shown in Fig. 3, the expression level in the fifth exon of the *APOB* gene drops abruptly and substantially in CDC cows.

**Figure 4:**
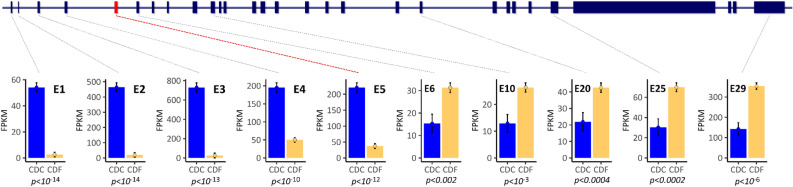
Exon-wise differential hepatic expression analysis of the *APOB* gene between CDC and CDF lactating cows. CDC denotes heterozygous carriers of the CD mutation; CDF animals are homozygous wild type. E-numbers indicate the analysed exons. Blue bars represent the expression levels of CDC animals; yellow bars represent those of CDF individuals. Indicated are lsmeans ± standard error. *p* values in italic represent the statistical significance of the differential expression between CDC and CDF. The red box in the gene model indicates the exon 5 affected by the LTR insertion.

In the liver of the studied cows, the read count level differed substantially between the section of the *APOB* gene upstream of the LTR insertion site in exon 5 and the region downstream of this site. Across exons 1–4, the expression was more than tenfold higher (FPKM 258.7 (se 15.89)) across CDC heterozygous carriers compared to wild-type animals (FPKM 21.4 (se 8.28), *p* = 7.113 e^−15^, Supplementary Figure 2). Specifically, the expression for each of the *APOB* exons upstream of the LTR retrotransposon insertion (exon 1–4) was significantly higher in CDC heterozygous carriers compared to wild-type animals (Fig. 4) including the alternatively spliced exon 2. This also applied to the expression of exon 5, which displayed the same statistically significantly higher expression in CDC compared to CDF animals as seen for exons 1–4 (*p* = 7.70 e^−13^, Fig. 4).

An expression pattern of *APOB* exons 1–5 similar to the one found in the studied cows was analogously observed in an independent study, which monitored the jejunal mucosa transcriptome of young calves^39^. CDC calves showed a specific drop of read coverage at the insertion point of the LTR retrotransposon (Supplementary Figure 3), and for exons 1–5, the CDC calves had a significantly higher expression than the CDF wild type calves (Supplementary Figure 4).

**Figure 5:**
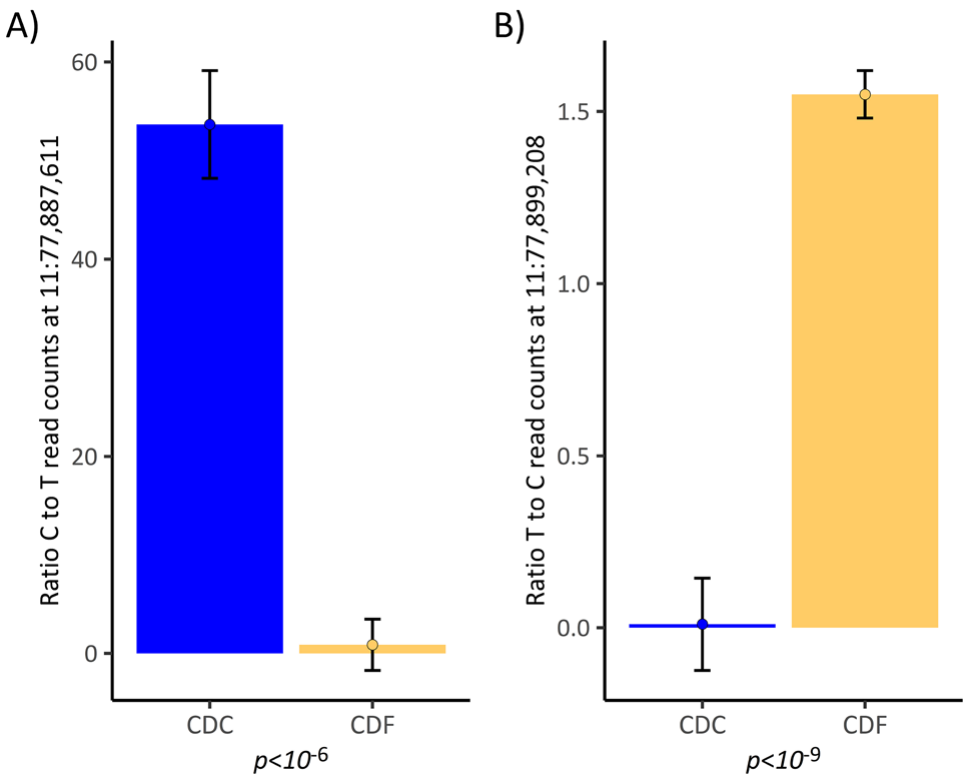
Ratio of reads mapping to alternative haplotypes for SNP positions in exon 3 (position 11:77,887,611) and exon 13 (position 11:77,998,208) of the *APOB* gene in CDC and CDF cows. (**A**) Ratio of C (allele on LTR insertion carrying haplotypes) to T (allele on the wild type haplotype in CDC animals), (**B**) ratio of T (allele on LTR insertion carrying haplotypes) to C (allele on the wild type haplotype in CDC animals). Indicated are lsmeans ± standard error.

Compared to the hepatic *APOB* expression pattern for exons 1–5, however, in the studied cows the situation was reversed for exons 6–29: the CDC animals displayed about only half the expression level of the CDF animals (Fig. 4). Assuming that the *APOB* gene expression was abolished downstream of the LTR insertion for the affected haplotype, the expression data for this part of the *APOB* gene would be consistent with the hypothesis that only one active gene copy is present in the CDC animals, or a premature polyadenylation signal was introduced by the ERV-K-LTR sequence insertion, as described by Harland^8^. The higher expression of the exons 1–4 could have resulted from a general activation of *APOB* transcription via a regulatory feedback loop in an attempt to compensate for the overall insufficient *APOB* expression. This would affect the transcription from both *APOB* gene copies. To evaluate a putative allele-biased expression and, thus, a potential direct regulatory effect of the LTR retrotransposon insertion, we monitored the number of reads covering SNPs in exon 3 (at position 11:77,887,611) upstream of the insertion and in exon 13 (at position 11:77,998,208) downstream of the insertion. From genotypes in variant positions that are located upstream, within and downstream of the *APOB* gene, which had been determined from Illumina HD genotype data in a half-sib offspring cohort from a CDC carrier sire, we could conclude that the C allele (alternative *APOB* allele to the ARS1.2 genome assembly) at position 11:77,887,611 was on the same haplotype as the LTR insertion in exon 5 of the *APOB* gene in CDC animals. Comparing the ratio of read counts for the C and the T allele in heterozygous animals at position 11:77,887,611, we found that the ratio for the CDF animals was close to the expected ratio of 1 ranging from 0.74 to 1.73 (lsmean 0.873, se 2.60, Fig. 5A). In contrast, the heterozygous CDC animals displayed a statistically significant (*p* = 8.863 e^-07^) higher ratio ranging from 35.0 to 82.27 (lsmean 53.669, se 5.46, Fig. 5A). This demonstrates a clear allele-biased expression of the *APOB* exon 3, which is located upstream of the LTR insertion.

We also performed the same analysis, which was conducted for position 11:77,887,611 (SNP in exon 3), also in the genomic region downstream of the LTR insertion for position 11:77,998,208 (SNP in exon 13). It has to be noted that the haplotype carrying the LTR retrotransposon insertion has a T (reference allele in the ARS1.2 reference genome assembly) in position 11:77,998,208. Compared to position 11:77,887,611, for 11:77,998,208, the ratio of reads from the two alternative haplotypes was in opposite direction in the heterozygous CDC animals (Fig. 5B). In the CDC animals, for 11:77,998,208, the number of read counts from the LTR carrying haplotype was much lower compared to the read counts from the wild type haplotype. Almost no reads were observed originating from the LTR retrotransposon-carrying haplotype. In contrast, the ratio of read counts for the CDF animals was much higher and closer to the expected ratio of 1. There was a highly significant difference in the ratio of read counts in CDC animals compared to the read count ratio for the two alternative haplotypes in the CDF cows (lsmean 0.0104, se 0.134, for CDC vs. lsmean 1.5498, se 0.069, for CDF, *p* = 1.583 e^−10^, Fig. 5B).

### Detecting genetic variants in the APOB genomic region

Whole genome resequencing obtained a coverage between 12.2 and 16.8 fold per sample. In the interval 11:77,385,988–77,891,739, we detected 3530 heterozygous or homozygous alternative allele positions for the six samples in our dataset. We wanted to explore if other variants than the LTR retrotransposon insertion could potentially be causal for the observed allele biased expression in exon 1–4 of the *APOB* gene. Any potential variant would have to be heterozygous in all CDC animals of our study, and the CDF animals, which did not show an allele biased expression, would have to be homozygous. In the genomic region upstream of the *APOB* transcription start site, no variant fulfilled this requirement until the boundaries of the next upstream neighbouring gene, *TDRD15*. The closest position to the *APOB* gene, which was heterozygous in all four CDC cows and homozygous in both CDF animals, was at 11:77,792,966, which is almost 100 kb distant from the *APOB* gene and upstream to the transcription start site of *TDRD15*.

Finally, we also inspected loci in the Farm Animal GTex dataset (^49^, https://cgtex.roslin.ed.ac.uk/downloads), which were described as *cis-*acting eQTL for the *APOB* gene. In liver tissue, no variant within 1 Mb upstream or downstream of the *APOB* gene showed a nominal *p* value < 10^–4^, and even those three with a *p*-value < 10^–3^ were located more than 200 kb downstream to the 3′ end of the *APOB* gene.

### Clinical and physiological effects of the CD carrier status

**Figure 6:**
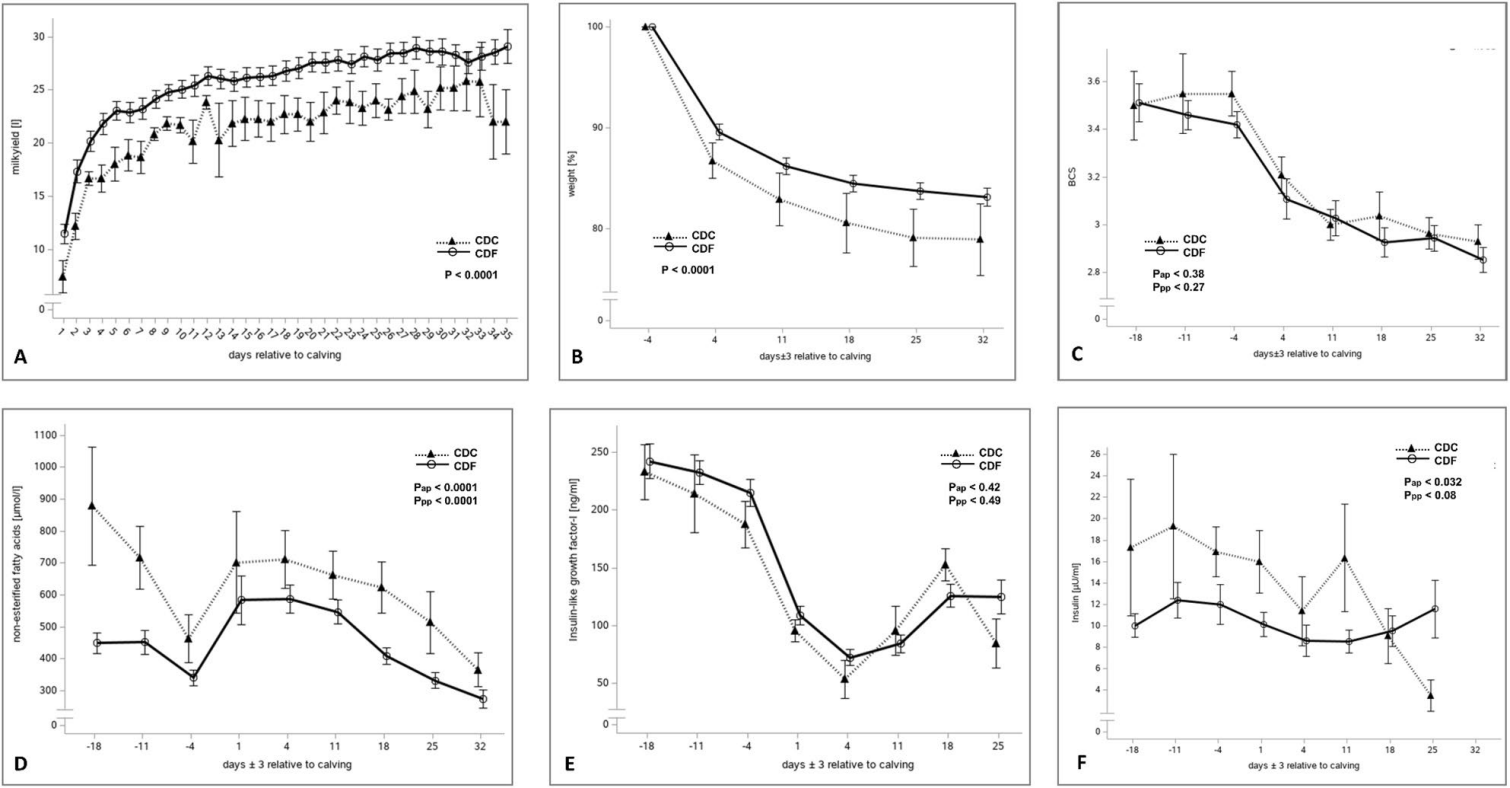
Clinical, biochemical and endocrinological characteristics of CDC cows compared to wild type CDF cows. (**A**) Energy-corrected milk, (**B**) body weight relative to weight, (**C**) body condition score, (**D)** blood serum concentration for non-esterified fatty acids, (**E**) blood plasma concentrations of IGF-1, (**F**) blood plasma concentrations of insulin. Data points indicate mean and standard error of the mean.

In the first 35 days of lactation, CDC animals had a significantly lower milk yield compared to CDF animals, but nevertheless showed a stronger mobilization of body mass indicated as loss of body weight after calving (Fig. 6A,B). This increased loss of body weight was not accompanied by a difference in the body condition score (Fig. 6C), which indicates an increased intraabdominal lipomobilization in CDC cows. For the CDC cows, increased lipomobilization known to be characterized by elevated NEFA levels^50^ was confirmed by a significantly higher level of NEFA in blood serum before and after calving (Fig. 6D). But given the decrease in body mass and a lower milk yield, the animals were presumably less capable of utilizing the mobilized resources. Despite the obvious differences in metabolism, there were no significant differences in IGF1 (Fig. 6E), GH and insulin level in blood plasma (Fig. 6F) after calving suggesting that the somatotropic axis of CDC cows was not directly affected by a copy of the LTR retrotransposon insertion *APOB* defect allele.

All three CDC cows challenged intramammary with *E. coli* developed acute clinical mastitis during the course of the animal experiment, while only four out of eight CDF cows showed a severe clinical response. Although the proportion of acute clinical mastitis cases was nominally higher in CDC compared to CDF animals, this difference was not statistically significant (*p* = 0.40). When challenged with *S. aureus*, no difference was seen in the incidence of clinical mastitis between the CDC (one out of four) and CDF group (three out of 20, *p* = 1).

## Discussion

The phenotypic data on the heterozygous carrier CDC animals as well as the results from the transcriptome analysis indicated a phenotypic effect from a single copy of the *APOB* gene carrying the LTR retrotransposon insertion. Across all exons of the *APOB* gene, we observed about 50% decreased expression in CDC carriers compared to the CDF homozygous wild type animals. This is in line with results from an abrogated *APOB* expression in homozygous CDS carriers^6–8^ and would be expected due to the lack of one copy of a fully functional gene. However, our data highlighted a strong discrepancy between the expression level for *APOB* exons upstream and downstream to the LTR retrotransposon insertion. The lower expression level for exons 6–29 downstream of the insertion compared to exons 1–5 could well be explained by the termination of transcription via a polyadenylation signal in the inserted LTR retrotransposon leading to the premature attachment of a poly(A) tail as described by Harland^8^. The first obvious explanation for the increased expression of exons 1–5 in the *APOB* gene would be a regulatory compensatory loop for the lack of an intact APOB protein resulting from the impaired expression of exons 6–29, which encode more than 95% of the APOB protein. However, a respective general compensatory mechanism should affect the expression regulation for both *APOB* copies regardless of the LTR retrotransposon insertion, e.g. via increased transcription factor activity or recruitment in the promotor region of the *APOB* gene^51^. To evaluate this hypothesis, we tested the expression output from both haplotypes separately. For the allele-biased expression analysis, we took benefit from variant positions in the coding part of the *APOB* gene as well as SNPs upstream and downstream of the gene body. After establishing the *APOB* haplotype carrying the LTR retrotransposon insertion, we were subsequently able to determine the haplotype origin of reads spanning variant positions in *APOB* exons 3 and 13.

The respective data in our study demonstrated that the enhanced expression of *APOB* exons 1–5 in CDC animals seems to be attributed to the haplotype carrying the *APOB* LTR retrotransposon insertion, which created a very distinct allelic expression bias. Our data were obtained via preparation of a stranded RNAseq library with poly(A) selection from RNA meticulously cleaned of any DNA contamination. This should avoid the issues with transposable element transcription detection due to pervasive transcription and provide reliable information on structure and source of transcripts, particularly with respect to transposable element molecules^10^.

**Figure 7:**
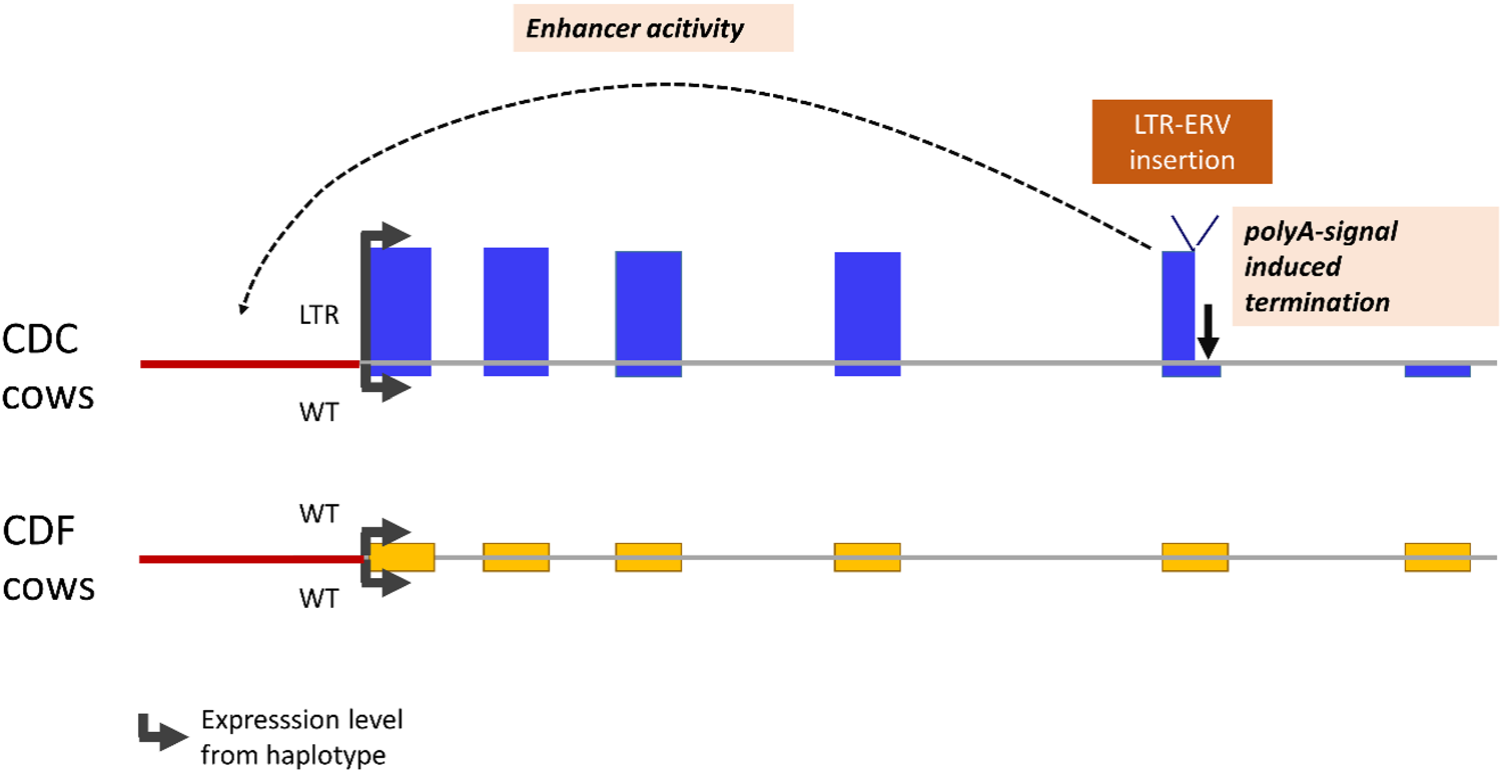
Model for modulated *APOB* expression due to ERV-K LTR insertion via combined enhancer activity and premature poly (A) signal insertion. Yellow and blue boxes indicate the expression levels for *APOB* exons 1–6 originating from the wild type (WT) haplotype or the haplotype with LTR insertion in CDC heterozygous carrier cows and homozygous CDF wild type cows. Red line indicates the promotor region of the *APOB* gene.

In summary, the *APOB* transcription data might be interpreted as a specific *cis-*regulatory enhancer effect exerted by the LTR retrotransposon insertion in the *APOB* gene (Fig. 7).

According to Harland^8^, the LTR retrotransposon inserted in exon 5 of the *APOB* gene belongs to the ERV2-1-BT_LTR group of the bovine endogenous retrovirus (ERV) family K. ERVKs comprise a subset of bovine retroviruses and seem to be currently active in the bovine genome^52^. For the human genome, HERV-K transposable elements have been found to be enriched with proximal and distal enhancer element motifs compared to the whole genome sequence^53^. Transposable elements, and LTR retrotransposons in particular, are known for their regulatory activities as reviewed by Chuong et al.^9^. The ERV-K LTR insertion in the bovine *APOB* gene comprises two full-length LTR flanking regions. Within LTRs, their U3 region is known to contain enhancer elements, which activate and drive viral transcription^54^ and can also modulate expression of nearby cellular genes^55^. LTRs are involved, e.g. in a regulatory dysfunction leading to leukaemia^56^ and are postulated for being responsible for ectopic CYP19A1 expression associated with the henny feathering phenotype in chicken^57^. Recently, the advances in CRISPR technology-enabled progress in experimental validation of the regulatory function of LTR retrotransposons^9^, which previously relied mainly on, e.g. selection signatures and chromatin features. Deniz et al.^56^ demonstrated via CRISPR-mediated targeted transposable element deletion in cancer cells that an LTR located proximal to the *APOC1* gene has an enhancer function for *APOC1* expression and also seems to exert a regulatory function on the downstream *APOE* gene. However, there are still very few studies demonstrating a regulatory function of LTR retrotransposons in vivo, particularly in natural models and non-model organisms. Our data suggest that the bovine *APOB* gene LTR retrotransposon insertion indeed is an in vivo model for a remarkable enhancer activity of ERV elements. Due to the widespread occurrence of LTR retrotransposon insertions, as reviewed by Thompson et al.^58^ and particularly due to the biological activity of the ERV-K type retroviruses^8, 59^, they might be a major source of diversity in gene regulation in the bovine genome.

Based on the gene expression data of our study alone, it could not be formally excluded that other variants on the LTR retrotransposon carrying haplotype could be responsible for the enhancer-like effects in our data set. To explore this option, we analysed transcriptomic and whole genome resequencing data from the neighbourhood of the *APOB* gene. No gene in the region comprising 2 Mb upstream or downstream of the *APOB* gene showed a significantly differential expression between CDC and CDF animals, which excludes an element with general enhancer activity in this region on the LTR insertion carrying haplotype. Since 500 kb upstream of the *APOB* gene, no other variant fulfilled the requirements of (i) heterozygosity in all resequenced CDC animals and (ii) homozygosity of the resequenced CDF animals, we conclude that other elements with specific *cis* activity for *APOB* expression regulation on the LTR insertion carrying haplotype are very unlikely.

The *APOB* gene is predominantly expressed in the liver and small intestine with the alternative predominant isoforms in the small intestine (APOB-48) and liver (APOB-100) as reviewed by Whitfield et al.^60^. This is confirmed by whole transcriptome RNAseq data analyses of blood and mammary gland samples from CDC and CDF cows in this study, which did not show indication of *APOB* gene expression (^61^, data not shown). The *APOB* gene is known for a tissue-specific posttranscriptional mRNA editing in the small intestine^62^, which creates a premature stop codon and is responsible for producing the truncated APOB-48 protein in intestine tissue. The signature of this regulatory editing process was also observed in the jejunal mucosa samples of our study (Supplementary Figure 5) and might have contributed to the divergent *APOB* expression pattern in the 3’ exons between CDC hepatic and intestinal samples relative to CDF samples (Fig. 4, Supplementary Figure 4). In the liver, the APOB-100 protein plays an essential role in the endocytosis of LDL complexes via a ligand function for the cellular LDL receptor. The smaller APOB-48 protein is required for building chylomicrons in the intestine and consequently for lipid uptake from the diet (reviewed by Whitfield et al.^60^). Due to its essential role in lipid metabolism, alterations in the *APOB* gene expression are likely to exert function beyond direct modulation of lipid intake.

Our transcriptomic data from the differential expression analysis confirm that the ERV-K LTR insertion indirectly affects the regulation of cholesterol synthesis in addition to its direct effect on *APOB* transcription. Earlier studies identified that CDC animals show a decreased plasma cholesterol level^11, 12^. Gross et al.^12^ suggested that this results from a lack of APOB protein with the consequence of building less HDL, LDL and/or VLDL complexes containing cholesterol. Thus, the authors postulated that the decreased blood cholesterol levels would not reflect the true cholesterol abundance in the CDC animals. However, hepatic transcriptomes of CDC animals in our study show that important genes encoding enzymes for the final step of cholesterol synthesis are significantly lower expressed compared to CDF wild type animals. Therefore, the decreased plasma cholesterol level in CDC animals carrying the LTR insertion might also result from a decreased cholesterol synthesis in addition to a lack of cholesterol transport capacity. Reduced cholesterol synthesis, as indicated by our data, could explain the detrimental effects observed in heterozygous CDC individuals as described by Häfliger et al.^13^. In addition, cholesterol is the starting point of estrogen biosynthesis, and our data indicate that also this biological pathway is significantly affected in CDC carriers, which could result in impaired reproduction of CDC carriers.

The phenotypic and the transcriptomic data of our study indicated an effect of a single copy of the LTR retrotransposon carrying the *APOB* gene. This is in line with reports from Häfliger et al.^13^ and confirms that the CD defect does not follow a fully recessive mode of inheritance. As indicated by the effects on milk yield, body weight loss, body condition score, and NEFA, the CDC animals seem to mobilize more internal abdominal fat, however, at a decreased performance level. In cattle, excess energy is primarily stored as triglycerides, which are stored in the adipose tissue and are mobilized when the energy intake does not meet the energy demand^63, 64^. NEFA is one of the most important blood indicators of lipomobilization in ruminants and an indicator of negative energy balance in postpartum dairy cattle^15, 65^. CDC animals have a significantly higher level of NEFA compared to CDF animals, but a significantly lower milk yield, whereas the BCS change is comparable between both groups. This suggests that the animals were possibly not fully capable to utilize the mobilized lipid resources.

There was no statistically significant difference in the incidence of clinical mastitis in CDC animals compared to CDF animals. However, the number of CDC animals challenged with *E. coli* (N = 3) or *S. aureus* (N = 4) was relatively small in comparison to the number of challenged CDF animals (N = 8 and N = 20, respectively), which could reduce the statistical power. To our knowledge, there is no study that compared the incidence of clinical mastitis between CDC and CDF animals, although Cole et al.^14^ reported a small favourable effect on genetic merit of the CD mutation carrying haplotype for somatic cell score, an indicator trait for mastitis incidence.

There is upcoming evidence for structural variants playing a substantial role in gene expression diversity^66^. This is in line with the in-vivo experimental data obtained in this study. The gene regulatory effects of the ERV-K LTR beyond the decreased transcript level downstream of its insertion in the *APOB* gene demonstrate the strong potential of mobile elements to modify gene expression and complex phenotypes. Even though Scott et al.^66^ found that mobile elements were only weakly enriched for eQTL signals, future investigations of functional diversity within populations should direct specific attention to this class of genetic variants and their potential regulatory effects, because mobile element catalogues and graph-based genome exploration^8, 67, 68^ are only starting to be available, particularly for non-model organisms.

In conclusion, using transcriptomic and whole-genome sequencing data in combination with clinical data of CDC and CDF cows under pathogen challenge during the early first lactation, we demonstrated a divergent allele-biased expression pattern of the *APOB* gene in CDC animals. This expression pattern might be a consequence of enhancer activity induced by the LTR insertion in the *APOB* gene, in addition to an already reported artificial polyadenylation signal within the insertion. Furthermore, the LTR insertion significantly alters the hepatic expression of genes involved in lipid metabolism and cholesterol biosynthesis. CDC animals showed an indication of impaired lipid utilization compared to CDF animals. In addition, the *APOB* gene LTR insertion might represent an in vivo model demonstrating the regulatory function of a genomic mobile element insertion on gene expression and subsequent consequences.

## Supplementary Information

Supplementary Figure 1.

Supplementary Figure 2.

Supplementary Figure 3.

Supplementary Figure 4.

Supplementary Figure 5.

Supplementary Table 1.

Supplementary Table 2.

Supplementary Table 3.

Supplementary Table 4.

Supplementary Table 5.

Supplementary Legends.

Supplementary Information.

## Data Availability

RNA-Seq datasets are publicly available from the ENA data portal (https://www.ebi.ac.uk/ena) (Project Numbers PRJEB33849, PRJEB24380). Whole genome resequencing data have been submitted to the ENA repository (https://www.ebi.ac.uk/ena) under Project Number PRJEB51478.
