# Supplementary material for: Allele-biased expression of the bovine APOB gene associated with the cholesterol deficiency defect suggests cis-regulatory enhancer effects of the LTR retrotransposon insertion
Source: Sci Rep. 2022 Aug 5;12:13469. doi: 10.1038/s41598-022-17798-5 (PMC9355974; doi:10.1038/s41598-022-17798-5)
Supplement: Supplementary file 1 — Supplementary Figure 1. [file 41598_2022_17798_MOESM1_ESM.htm]

Pathway


|  |  |
| --- | --- |
| **Pathway:Steroid hormone biosynthesis** |  |
|  |  |
| **Pathway information generated by KEGG.        Stop Blinking** |  |
|  |  |
| **|  | | --- | |  | | **List genes are shown in red** | | |  | | --- | | **DAVID Gene Name** | | UDP glucuronosyltransferase 1 family, polypeptide A1(UGT1A1) | | UDP glucuronosyltransferase 1 family, polypeptide A6(UGT1A6) | | UDP glucuronosyltransferase 2 family(MGC152010) | | UDP glucuronosyltransferase 2 family, polypeptide B10(UGT2B10) | | UDP glucuronosyltransferase family 2 member A1 complex locus(UGT2A1) | | UDP-glucuronosyltransferase 2B17(LOC100140261) | | UDP-glucuronosyltransferase 2B31(LOC100138004) | | UDP-glucuronosyltransferase 2B4(LOC100138908) | | UDP-glucuronosyltransferase 2B4(LOC100296421) | | UDP-glucuronosyltransferase 2B4(LOC540615) | | UDP-glucuronosyltransferase 2B4(LOC615303) | | UDP-glucuronosyltransferase 2C1(LOC530553) | | aldo-keto reductase family 1 member D1(AKR1D1) | | catechol-O-methyltransferase(COMT) | | catechol-O-methyltransferase(COMT) | | cytochrome P450 11B1, mitochondrial(LOC787628) | | cytochrome P450 2B4-like(LOC530571) | | cytochrome P450 2C31(LOC785540) | | cytochrome P450 subfamily 2B(CYP2B6) | | cytochrome P450, family 1, subfamily A, polypeptide 2(CYP1A2) | | cytochrome P450, family 1, subfamily B, polypeptide 1(CYP1B1) | | cytochrome P450, family 11, subfamily A, polypeptide 1(CYP11A1) | | cytochrome P450, family 17, subfamily A, polypeptide 1(CYP17A1) | | cytochrome P450, family 19, subfamily A, polypeptide 1(CYP19A1) | | cytochrome P450, family 2, subfamily C, polypeptide 18(CYP2C18) | | cytochrome P450, family 2, subfamily C, polypeptide 87(CYP2C87) | | cytochrome P450, family 2, subfamily D, polypeptide 6(CYP2D14) | | cytochrome P450, family 2, subfamily E, polypeptide 1(CYP2E1) | | cytochrome P450, family 2, subfamily c(LOC511498) | | cytochrome P450, family 21, subfamily A, polypeptide 2(CYP21A2) | | cytochrome P450, family 3, subfamily A, polypeptide 5(CYP3A5) | | cytochrome P450, family 7, subfamily A, polypeptide 1(CYP7A1) | | cytochrome P450, family 7, subfamily B, polypeptide 1(CYP7B1) | | cytochrome P450, subfamily I (aromatic compound-inducible), polypeptide 1(CYP1A1) | | cytochrome P450, subfamily IIIA (niphedipine oxidase), polypeptide 4(CYP3A5) | | cytochrome P450, subfamily IIIA, polypeptide 4(CYP3A4) | | cytochrome P450, subfamily XI B, polypeptide 1(CYP11B1) | | cytochrome P450, subfamily XXI (steroid 21-hydroxylase)(CYP21) | | dihydrodiol dehydrogenase 3(LOC100337056) | | hydroxy-delta-5-steroid dehydrogenase, 3 beta- and steroid delta-isomerase 1(HSD3B1) | | hydroxysteroid (17-beta) dehydrogenase 6(HSD17B6) | | hydroxysteroid 11-beta dehydrogenase 1(HSD11B1) | | hydroxysteroid 11-beta dehydrogenase 2(HSD11B2) | | hydroxysteroid 17-beta dehydrogenase 1(HSD17B1) | | hydroxysteroid 17-beta dehydrogenase 12(HSD17B12) | | hydroxysteroid 17-beta dehydrogenase 2(HSD17B2) | | hydroxysteroid 17-beta dehydrogenase 3(HSD17B3) | | hydroxysteroid 17-beta dehydrogenase 7(HSD17B7) | | hydroxysteroid 17-beta dehydrogenase 8(HSD17B8) | | steroid 17-alpha-hydroxylase/17,20 lyase(LOC101908425) | | steroid 5 alpha-reductase 1(SRD5A1) | | steroid 5 alpha-reductase 2(SRD5A2) | | steroid 5 alpha-reductase 3(SRD5A3) | | steroid sulfatase (microsomal), isozyme S(STS) | | sulfotransferase family 1E member 1(SULT1E1) | | sulfotransferase family 2B member 1(SULT2B1) | | uncharacterized LOC785824(MGC127055) | | |  | |  |** |
