# Supplementary figures and images for: Allele-biased expression of the bovine APOB gene associated with the cholesterol deficiency defect suggests cis-regulatory enhancer effects of the LTR retrotransposon insertion
Source: Sci Rep. 2022 Aug 5;12:13469. doi: 10.1038/s41598-022-17798-5 (PMC9355974; doi:10.1038/s41598-022-17798-5)

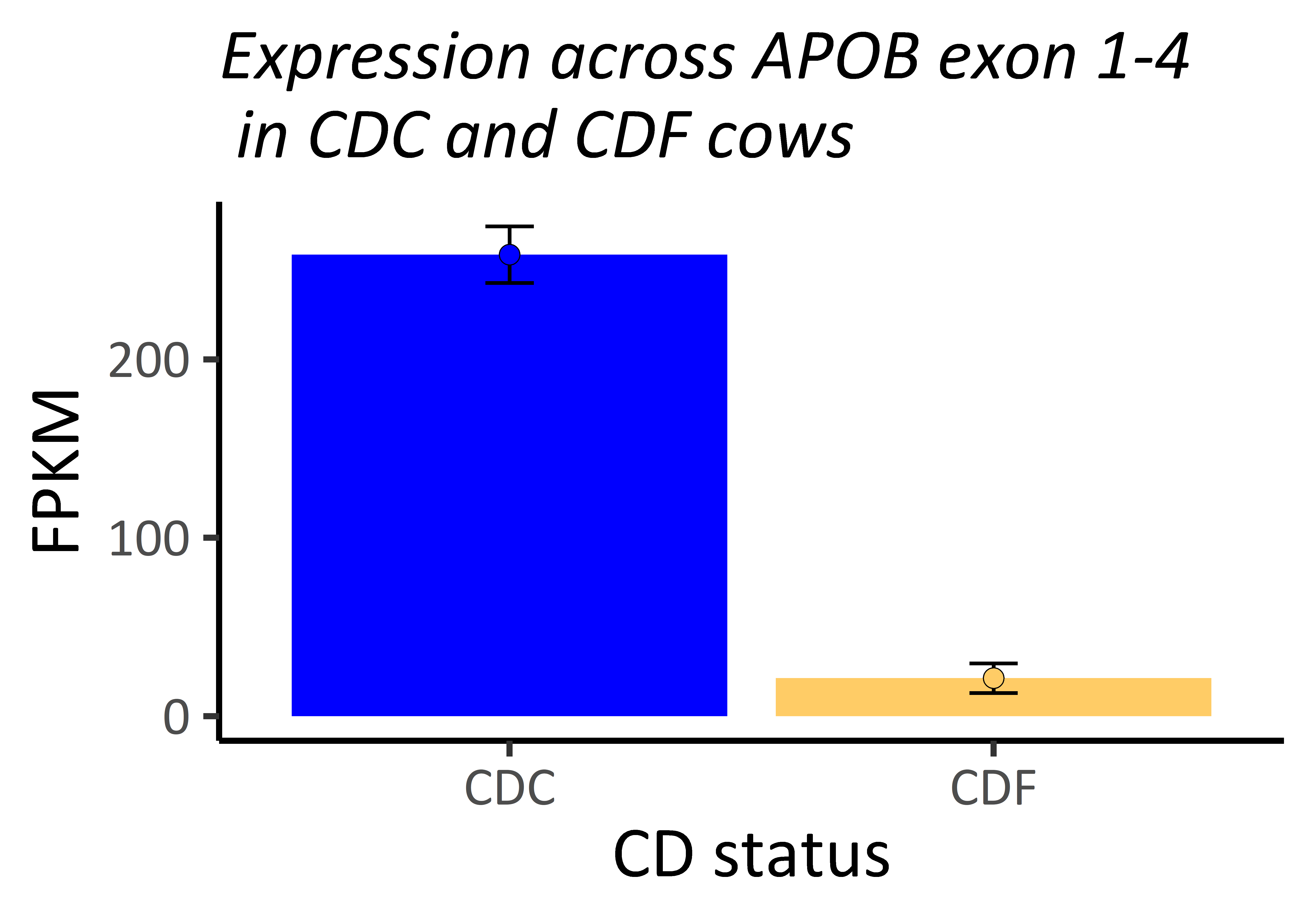

Supplement: Supplementary file 2 — Supplementary Figure 2. [file 41598_2022_17798_MOESM2_ESM.tiff]

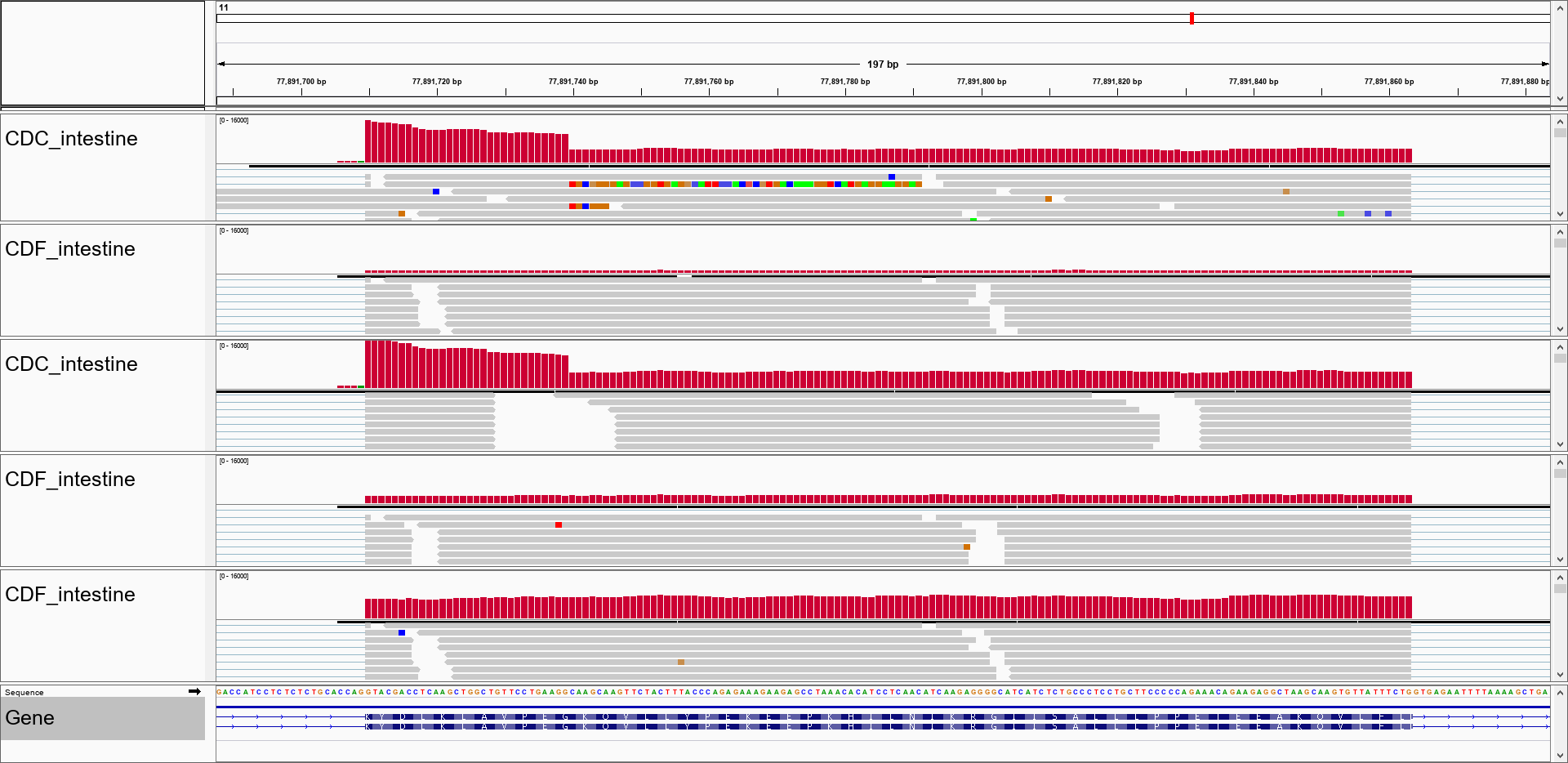

Supplement: Supplementary file 3 — Supplementary Figure 3. [file 41598_2022_17798_MOESM3_ESM.png]

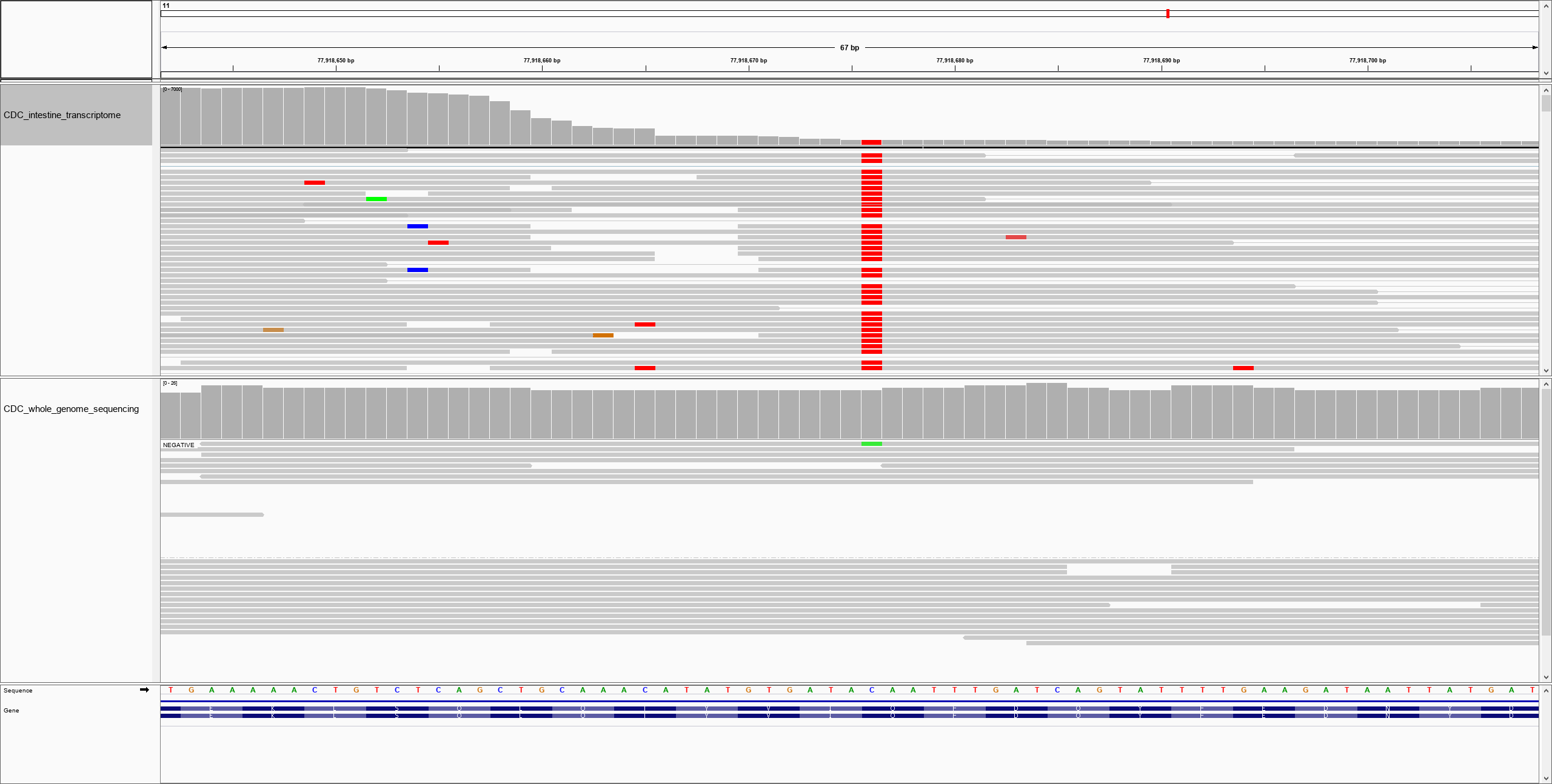

Supplement: Supplementary file 5 — Supplementary Figure 5. [file 41598_2022_17798_MOESM5_ESM.png]
