# Supplementary material for: Allele-biased expression of the bovine APOB gene associated with the cholesterol deficiency defect suggests cis-regulatory enhancer effects of the LTR retrotransposon insertion
Source: Sci Rep. 2022 Aug 5;12:13469. doi: 10.1038/s41598-022-17798-5 (PMC9355974; doi:10.1038/s41598-022-17798-5)
Supplement: Supplementary file 11 — Supplementary Legends. [file 41598_2022_17798_MOESM11_ESM.docx]

# Supporting information legends

**Suppl. Fig. 1: KEGG Steroid hormone biosynthesis pathway showing enrichment of genes differentially expressed in the hepatic transcriptome between CDC and CDF cows**

**Suppl. Fig. 2: Histogram of FPKM values for *APOB* gene exon 1-4 expression comparing heterozygous CDC carriers and CDF non-carriers**. FPKM: Fragments per kilobase of transcripts per million mapped reads. (lsmeans ± standard error.

**Suppl. Fig. 3: Screenshot of *APOB* exon 5 jejunum expression profiles the Integrative genomics viewer**. Unaffected CDF calves and heterozygous CDC calves

**Suppl. Fig. 4: Exon-wise differential jejunal mucosa expression analysis of the *APOB* gene between CDC and CDF young calves cows**. CDC denotes heterozygous carriers of the CD mutation (N=7); CDF animals are homozygous wild type (N=4). E-numbers indicate the analysed exons. Blue bars represent the expression

**Suppl. Fig. 5: Screenshot of *APOB* position BTA11:77,918,676.** IGV image of the jejunum expression profile and profile from whole genome resequencing of an affected heterozygous CDC calf. Posttranscriptional editing responsible for generating the apoB48 protein isoform is indicated by the epimutation in the transcript profile

**Suppl. Text 1: Pipeline for whole genome resequencing data analysis**

**Suppl. Table 1: Primer sequences and annealing temperature for amplification and resequencing of APOB gene exons 3 and 13**

**Suppl. Table 2: Results from differential expression analysis between CDF and CDC cows from hepatic transcriptome resequencing.** Gene_id: Gene identifier according the Ensembl, baseMean: normalized expression mean across samples as estimated by DESeq2, lfcSE: standard error of the log2FoldChange, padj: p-value after adjustment for multiple testing

**Suppl. Table 3: List of KEGG and REACTOME pathways and Gene Ontology terms significantly enriched with genes differentially expressed in the hepatic transcriptome of CDC and CDF cows.**

**Suppl. Table 4: List of Ingenuity canonical pathways significantly enriched with genes differentially expressed in the hepatic transcriptome of CDC and CDF cows.**

**Suppl. Table 5: List of upstream regulators of hepatic differential gene expression between CDC and CDF cows as predicted from Ingenuity Pathway analysis**
